# The effect of vitamin K on prothrombin time in critically ill patients: an observational registry study

**DOI:** 10.1186/s40560-020-00517-5

**Published:** 2021-01-18

**Authors:** Sofia Dahlberg, Ulf Schött, Thomas Kander

**Affiliations:** 1 Division of Anaesthesia and Intensive Care, Department of Clinical Sciences, Lund University, SE-22184 Lund, Sweden; 2 Department of Anaesthesia and Intensive Care, Skane University Hospital Lund, Lund, Sweden

**Keywords:** Coagulopathy, Bleeding, Prothrombin time, PT-INR, Vitamin K, Intensive care

## Abstract

**Background:**

Previous studies have indicated that vitamin K deficiency is common in non-bleeding critically ill patients with slightly prolonged prothrombin time-international normalized ratio (PT-INR). It has never been investigated thoroughly whether the administration of vitamin K to these patients could affect their PT-INR. Therefore, the aim of this registry study was to evaluate changes in PT-INR in response to vitamin K in critically ill patients with PT-INR in the range of 1.3–1.9.

**Methods:**

Patients admitted to a mixed 9-bed general intensive care unit at a University Hospital, between 2013 and 2019 (*n* = 4541) with a PT-INR between 1.3 and 1.9 at any time during the stay were identified. Patients who received vitamin K with appropriate sampling times for PT-INR and without exclusion criteria were matched with propensity score to patients from the same cohort who did not receive vitamin K (controls). PT-INR was measured at admission, within 12 h before vitamin K administration and 12–36 h following vitamin K administration. Exclusion criteria included pre-existing liver cirrhosis, any plasma or platelet transfusion, or > 1 unit red blood cell transfusion between PT-INR samplings.

**Results:**

Propensity score matching resulted in two groups of patients with 129 patients in each group. PT-INR decreased in both groups (1.4 [1.3–1.4] in the vitamin K group and 1.4 [1.3–1.6] in the controls, *p* < 0.001 and *p* = 0.004, respectively). The decrease in PT-INR was slightly more pronounced in patients who received vitamin K (delta PT-INR − 0.10 [− 0.30 to − 0.10] in the vitamin K group and − 0.10 [− 0.20 to 0.10] in the controls, *p* = 0.01).

**Conclusion:**

In critically ill patients with a PT-INR of 1.3–1.9, the administration of vitamin K resulted in a slightly larger decrease of PT-INR 12–36 h after administration compared to controls. Future studies should focus on identifying which patient populations may benefit most from vitamin K administration as well as whether vitamin K could be a better alternative than plasma or prothrombin complex concentrate to improve PT-INR before non-emergent invasive procedures.

## Introduction

Coagulopathies are frequently present in critically ill patients, and they contribute to increased mortality [1, 2]. Increased bleeding diathesis may be corrected in several ways, depending on the severity and the patient’s circumstances, and vitamin K may be used as a sole therapy or in combination with other pro-coagulant agents [3, 4]. However, the current evidence is limited regarding the effect of intravenous vitamin K on standard coagulation assays in critically ill patients, and it is not clear which dose and administration interval should be used. Furthermore, patients with mild coagulopathy are often given prophylactic plasma before invasive procedures albeit low evidence and risks associated with transfusion [5, 6]. Vitamin K might be a preferable option in this setting. Much of current knowledge of intravenous vitamin K pharmacology relates to its use as a warfarin reversal drug alone or together with plasma/prothrombin complex concentrates [7]. The PT-INR has a clear role in warfarin-treated patients to monitor bleeding and thrombosis risks and the effect of corrective treatment with vitamin K [8]. PT as a bleeding risk indicator in the non-anticoagulated patient before liver and renal biopsies, central venous catheterization and tracheostomy are not that clear-cut [9–12]. However, patients with haemothorax and chest tube drainage have increased mortality if PT-INR > 1.6 [13].

A previous study has estimated the prevalence of vitamin K deficiency to be ~ 25%, with a peak incidence at intensive care unit (ICU) admission [14]. Whether vitamin K deficiency is dangerous or not is still unclear in non-bleeding situations, but it is well known that vitamin K has many other positive effects beyond its indisputable role in coagulation. For example, vitamin K acts as a cofactor for vitamin K-dependent proteins, which are involved in cardiovascular health, bone metabolism and cancer [15].

In a recent study from our department, we have demonstrated that patients admitted to the ICU have increased levels of hypocarboxylated prothrombin “protein induced by vitamin K absence or antagonism for factor II” (PIVKA-II) indicating sub-clinical vitamin K deficiency at admission, and that this deficiency further increases during the ICU stay [16]. Although a small previous study demonstrated some changes in PT-INR when vitamin K was given to selected critically ill patients [14], the role of vitamin K in non-bleeding critically ill patients is largely unknown. In an attempt to further study the effect of intravenously given vitamin K on PT-INR, we matched patients by propensity score with mild to moderate prolonged PT-INR who were given vitamin K to those who were not given vitamin K in a large consecutive cohort of patients from a single ICU. Our hypothesis was that PT-INR would decrease more in patients who received vitamin K than in those who did not.

## Methods

### Study design and population

This single-centre, registry study was approved by the Regional Ethical Review Board in Lund, Sweden (registration numbers 2014/916 and 2018/866), and was conducted in compliance with the Declaration of Helsinki. The study was performed at a nine-bed general ICU at Lund University Hospital from September 2013 to May 2019, and the manuscript was written according to the STROBE guidelines for observational studies [17].

PT analysis was performed using the Owren PT-INR, which is calibrated using reference plasma samples from Equalis (Uppsala, Sweden). The normal reference range is 0.90–1.20, with a coefficient of variation of < 5%. The Owren method is only affected by variations of coagulation factors (f) II, VII and X, as compared to Quick-PT also affected by fI and fV.

All patients who were admitted to the ICU and had a PT-INR in the range of 1.3–1.9 at any time during their stay were identified in the registry. If the patient was given intravenous (iv) vitamin K1 (synthetic phytomenadione, Konakion Novum®, Cheplapharm Arzneimittel GmbH Ziegelhof 24, Greifswald, Germany), PT-INR had to be analysed < 12 h (h) before vitamin K administration (pre-treatment value) and 12–36 h following vitamin K administration (post-treatment value); otherwise, the case was excluded. Further exclusion criteria included any plasma- or platelet transfusion or a > 1 unit erythrocyte concentrate transfusion between PT-INR samplings and vitamin K administration within 72 h prior to the first PT-INR sampling. Patients with known liver cirrhosis were excluded from the study, as these patients may have prolonged PT-INR related to decreased synthesis of clotting factors. The administration of vitamin K was given at the discretion of the treating physician due to the absence of local guidelines.

For comparison, patients with PT-INR in the range of 1.3–1.9 not given vitamin K were included. These were defined as patients with prolonged PT-INR at their initial sampling (pre-treatment) with a follow-up sample within 12–36 h (post-treatment). The groups are referred to as the Vitamin K (VK) group and the control group respectively.

**Fig. 1:**
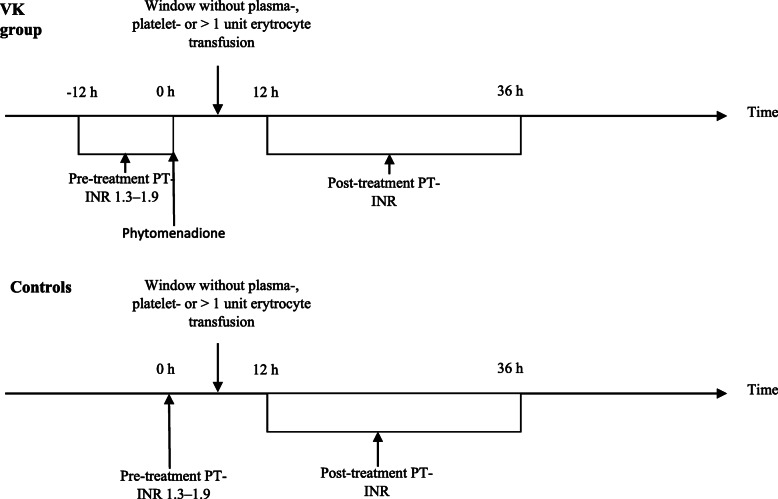
Schematic of study protocol. VK, vitamin K; PT-INR, prothrombin time, international normalized ratio

A schematic flow chart of the study design is shown in Fig. 1.

### Clinical data

The registry was based on laboratory test results, gender, age and transfusion data between sampling occasions, which were obtained from the patient data management system (ICCA, Philips, Amsterdam, The Netherlands). Additional data concerning patient comorbidities, reasons for admission, ICU severity scores and mortality were extracted from the local quality registry (PASIVA, Otimo Data AB, Kalmar, Sweden).

Comorbidities were reported in accordance with the Swedish Intensive Care Registry. The following categories of comorbidities were tracked: congestive heart failure, cancer, haematological malignancy and immunosuppressive therapy. Definitions for each category are shown in Table 1.

**Table 1:** Patient characteristics, labs and vital parameters expressed as median (interquartile range [IQR]) or no (percentage (%))

	Unmatched groups				Matched groups			
	Controls ***n*** = 615	VK group ***n*** = 134	Standardized difference	***p*** value	Controls ***n*** = 129	VK group ***n*** = 129	Standardized difference	***p*** value
**Variables included in matching**								
**SAPS 3 EMR**	24 (10–45)	26 (14–45) (20.8)	0.033	0.38	28 (12–47)	26 (14–42)	− 0.088	0.64
**Age (years)**	67 (54–75)	68 (55–75)	− 0.004	0.99	68 (54–75)	67 (55–74)	− 0.041	0.62
**Pre-treatment PT-INR**	1.3 (1.3–1.4)	1.5 (1.4–1.6)	0.78	< 0.001	1.5 (1.4–1.6)	1.5 (1.4–1.6)	0.005	0.96
**Sex (female)**	204 (33)	43 (30)	− 0.023	0.81	43 (33)	42 (33)	− 0.017	0.90
**Baseline variables not included in matching**								
**Reasons for admission**^**a**^**,** ***n*** **(%)**								
Severe sepsis or septic shock^b^	159 (26)	53 (40)	NA	0.001	53 (41)	52 (40)	NA	0.90
Trauma	61 (10)	16 (12)	NA	0.49	4 (3)	15 (12)	NA	0.009
CNS	254 (41)	33 (25)	NA	< 0.001	32 (25)	31 (24)	NA	0.89
Haematologic	41 (7)	9 (7)	NA	0.99	8 (6)	8 (6)	NA	1.0
Gastric	103 (17)	38 (28)	NA	0.002	31 (24)	38 (29)	NA	0.33
Metabolic	100 (16)	17 (13)	NA	0.30	23 (18)	17 (13)	NA	0.30
Respiratory	301 (49)	72 (54)	NA	0.32	69 (53)	69 (53)	NA	1.0
Cardiovascular	226 (37)	28 (21)	NA	< 0.001	51 (40)	27 (21)	NA	0.001
Hepatic	30 (5)	9 (7)	NA	0.39	7 (5)	9 (7)	NA	0.61
Renal	130 (21)	27 (20)	NA	0.79	38 (29)	27 (21)	NA	0.12
Other	50 (8)	15 (11)	NA	0.26	12 (9)	14 (11)	NA	0.68
**Comorbidities,** ***n*** **(%)**^**c**^								
Cancer^d^	85 (14)	26 (19)	NA	0.099	15 (12)	23 (18)	NA	0.15
Congestive heart failure^e^	30 (5)	9 (7)	NA	0.39	10 (8)	8 (6)	NA	0.63
Haematological malignancy^f^	22 (4)	6 (4)	NA	0.62	10 (8)	6 (5)	NA	0.31
Immunosuppressive therapy^g^	35 (6)	6 (4)	NA	0.58	8 (6)	6 (5)	NA	0.59
Multiple comorbidities	13 (2)	3 (2)	NA	0.93	2 (2)	3 (2)	NA	0.65

^a^Patients could have multiple reasons for admission that individually would justify intensive care

^b^According to the Sepsis 2 definition

^**c**^Patients could have multiple comorbidities

^d^Cancer disseminated beyond regional lymph nodes

^e^New York Heart Association stadium IV. Fatigue, dyspnoea or anginal pain at rest or minimal physical activity, which increases with physical activity

^f^Diagnosis of lymphoma, acute leukaemia or myeloma

^g^Systemic steroid treatment corresponding to at least 0.3 mg/kg of prednisolone daily within 6 months; external radiotherapy against invasive malignancy within 6 months; or chemotherapy due to malignancy, vasculitis, rheumatoid arthritis or inflammatory bowel disease

Severe sepsis and septic shock were classified according to the Sepsis 2 definition [18] because the majority of the data were collected from a time period prior to the establishment of the Sepsis 3 diagnosis criteria.

The expected mortality rate (EMR) calibrated for Swedish ICUs was computed based on the simplified acute physiology score (SAPS3) via a formula previously described [19].

Mortality was measured at 30, 90 and 180 days counting from ICU admission.

### Statistical analysis

Data were analysed using IBM SPSS for Windows, version 26.0 (SPSS Inc., Chicago, Ill., USA), and R, version 3.5.0 (Auckland, New Zealand).

To compensate for differences between the groups in selected baseline variables, propensity score matching was used. The covariates used for matching were age, gender, admission SAPS 3 EMR and pre-treatment PT-INR. The software used for propensity score matching combined the SPSS interface with calculations in R (Auckland, New Zealand) [20]. The propensity score was generated through logistic regression and a 1:1 nearest neighbour algorithm. To ensure a good match, a maximum allowable difference between two participants (caliper) was used. If no match was found, the case was excluded. To test the hypothesis and investigate whether vitamin K affected the magnitude of change in the PT-INR, delta values, defined as the difference between post- and pre-treatment PT-INR for the VK group versus the control group, were compared using the Mann-Whitney *U* test. Repeated measures were analysed using paired testing. Nominal data (gender, reasons for admission, comorbidities, mortality) were analysed using the chi-square test. Continuous variables are presented as a median (interquartile range [IQR]), and all categorical variables are presented as numbers (percentages). The statistical significance level was set to *p* < 0.05.

## Results

### Patient characteristics and baseline data

**Fig. 2:**
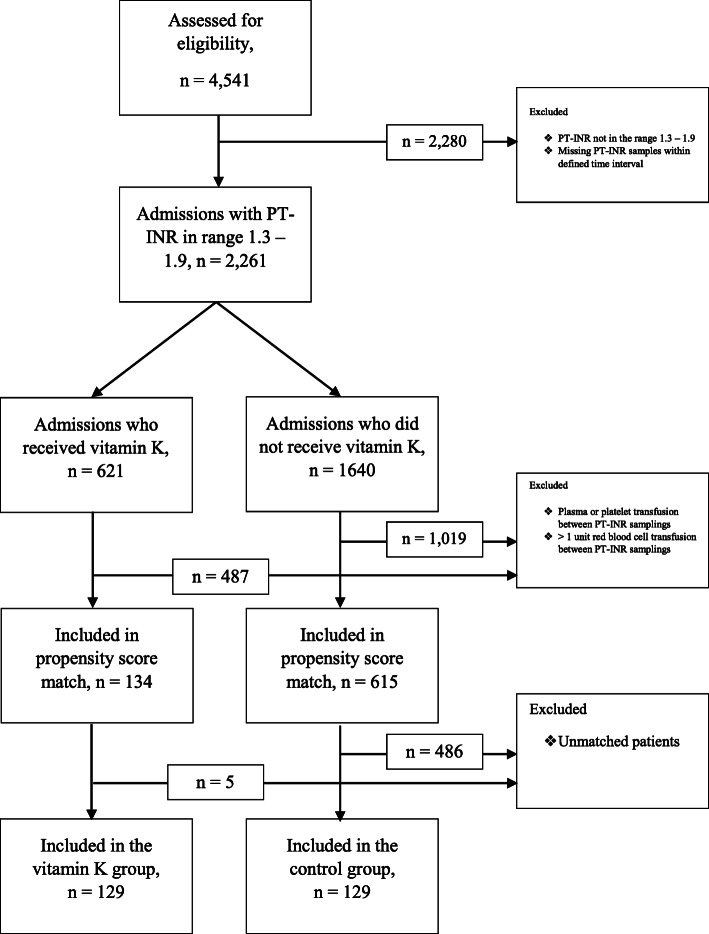
Consort diagram. PT-INR, prothrombin time, international normalized ratio

The groups were extracted from a database comprising 4541 records. A consort diagram showing the enrolment of patients is shown in Fig. 2.

In total, 621 patients with a pre-treatment PT-INR in the range of 1.3–1.9 received iv vitamin K. After applying exclusion criteria, 134 patients remained in the VK group. Vitamin K doses ranged from 5 to 20 mg, with 10 mg being the most commonly used dose (128/134, 96%).

The control group was identified from the same dataset. For that group, 1640 admissions with a PT-INR in the range 1.3–1.9 with repeated PT-INR samplings within 12–36 h who had not been given vitamin K were identified. After applying exclusion criteria, 615 patients remained in the control group. The propensity score-match resulted in 129 patients in the VK-group and 129 patients in the control group. Matching reduced the standardized difference to less than 10% for all included variables, indicating a good match [21].

For details on the baseline characteristics, propensity scores and standardized differences, please see Table 1. After the matching, two of the variables not included in the propensity score match differed between the groups. The prevalence of trauma as reason for admission was higher in the VK group compared to the control group (12% versus 3%, *p* = 0.009). By contrast, cardiovascular reason for admission was less common in the VK group compared to the control group (21% versus 40%, *p* = 0.001).

### Delta-changes

The median delta decrease of PT-INR was greater in the VK-group − 0.10 (− 0.30 to − 0.10) as compared to the control group − 0.10 (− 0.20 to 0.10) (*p* = 0.01). Delta values are shown in Table 2.

**Table 2:** Delta values, defined as the difference between post- and pre-treatment value. Values expressed as median (IQR)

Laboratory analyses	VK group ***n*** = 129	Controls ***n*** = 129	***p*** value
PT-INR	− 0.10 (− 0.10 to − 0.30)	− 0.10 (− 0.20 to 0.10)	0.01
APTT (s)	0 (− 3 to 4)	0 (− 4 to 5)	0.38
Platelets (× 10^9^/L)	− 8 (− 32 to 13)	− 11 (− 49 to 8)	0.12
Haemoglobin (g/L)	0 (− 3 to 2)	− 4 (− 12 to 3)	0.002
Fibrinogen (g/L)	0.5 (0 to 1.4)	0.2 (− 0.2 to 1)	0.023
CRP (mg/L)	17 (− 14 to 77)	25 (− 4 to 86)	0.34
Leukocytes (× 10^9^/L)	0 (− 2 to 2)	0 (− 3 to 2)	0.31

*VK* vitamin K, *PT-INR* prothrombin time, international normalized ratio, *APTT* activated partial thromboplastin time, *CRP* C-reactive protein

### Changes in response to vitamin K

**Fig. 3:**
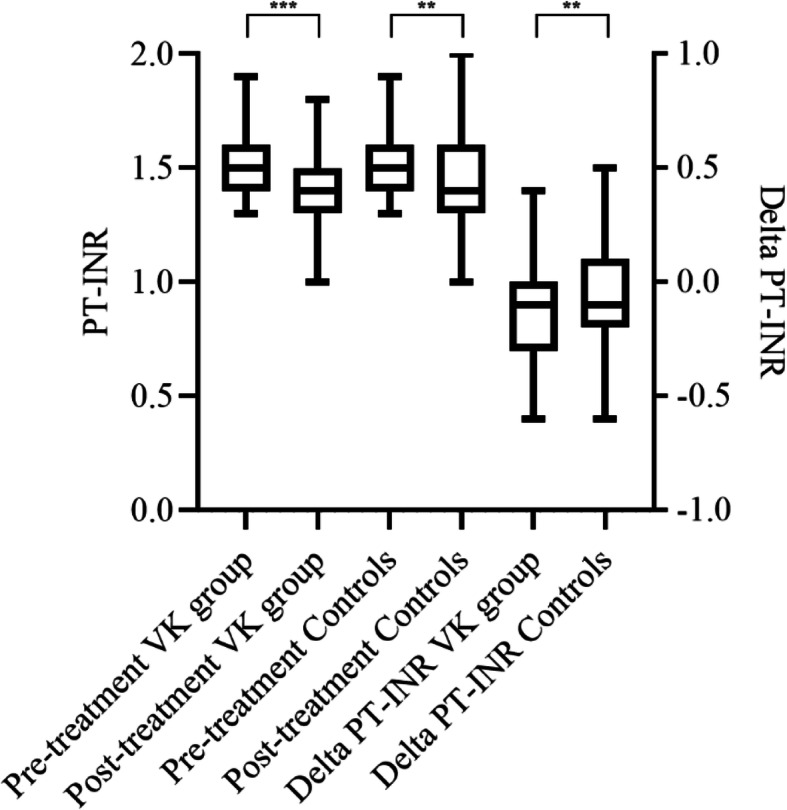
Boxplot of changes in PT-INR and delta PT-INR. Whiskers represent maximum and minimum values up to 1.5*IQR. Outliers were defined as > 1.5*IQR and are removed from the picture but were included in the calculations. VK, vitamin K; PT-INR, prothrombin time, international normalized ratio. **p ≤ 0.01, ***p < 0.001

The median pre-treatment PT-INR was 1.5 (1.4–1.5) in the VK group and 1.5 (1.4–1.6) in the control group (N.S.). At 12–36 h after the administration of vitamin K, the median PT-INR had decreased to 1.4 (1.3–1.4) (*p* < 0.001) in the VK group and to 1.4 (1.3–1.6) in the control group (*p* = 0.004) (Fig. 3 and Table 3). Changes in additional laboratory analyses are presented in Table 3.

**Table 3:** Laboratory analyses pre- and post-treatment. Values expressed as median (IQR)

Laboratory analyses	VK group pre-treatment ***n*** = 129	VK group post-treatment ***n*** = 129	Trend VK group	***p*** value VK group	Controls pre-treatment ***n*** = 129	Controls post-treatment ***n*** = 129	Trend controls	***p*** value controls
PT-INR	1.50 (1.40–1.60)	1.40 (1.30–1.50)	↓	< 0.001	1.5 (1.4–1.6)	1.4 (1.3–1.6)	↓	0.004
APTT (s)	35 (30–42)	34 (30–42)	→	0.96	36 (31–41)	34 (30–41)	→	0.41
Platelets (× 10^9^/L)	158 (121–224)	150 (103–221)	↓	0.008	167 (104–250)	153 (99–209)	↓	< 0.001
Haemoglobin (g/L)	102 (93–113)	102 (94–112)	→	0.19	108 (95–119)	103 (94–116)	↓	0.002
Fibrinogen (g/L)	4 (2.9–4.6)	4.6 (3.3–5.9)	↑	< 0.001	3.3 (2.3–4.9)	3.8 (2.9–5.1)	↑	< 0.001
CRP (mg/L)	146 (62–245)	181 (106–278)	↑	< 0.001	89 (35–183)	132 (61–232)	↑	< 0.001
Leukocytes (× 10^9^/L)	13 (9–18)	13 (9–18)	→	0.91	12 (8–17)	11 (9–16)	→	0.24

*VK* vitamin K, *PT-INR* prothrombin time, international normalized ratio, *APTT* activated partial thromboplastin time, *CRP* C-reactive protein

### Mortality

There were no difference in mortality between the VK group and the controls at 30 (*n* = 37 versus *n* = 35, *p* = 0.78), 90 (*n* = 51 versus *n* = 50, *p* = 0.90) and 180 days (*n* = 63 versus *n* = 58, *p* = 0.53) respectively.

## Discussion

In this retrospective observational study, the hypothesis that critically ill patients with prolonged PT-INR in the range of 1.3–1.9 who are given a single dose of vitamin K would show a larger decrease of PT-INR compared to the controls was proven true. However, the observed differences were relatively small and the clinical significance is unknown. It is possible that vitamin K administration may reinforce the normalization of a spontaneously prolonged PT-INR in critically ill patients.

Previous data on the natural course of a prolonged PT-INR in ICU patients is scarce. In a previous study on critically ill patients, we measured PT-INR and PIVKA-II over time [16]. PT-INR remained unchanged throughout, whereas PIVKA-II increased—a sign of sub-clinical vitamin K deficiency. This is in agreement with previous observations after major gastrointestinal surgery, where PIVKA-II also increased over time with unchanged PK-INR [22].

Notably, even though the administration of vitamin K resulted in a larger decrease in PT-INR than in controls, the difference in absolute numbers was relatively small. Based on these results, the clinical benefit of vitamin K administration in this population may be questioned. However, it could be argued that critically ill patients have small margins and are at constant risk for deterioration, wherefore even small improvements should be encouraged. Also, it is possible that the administration of vitamin K protects patients from developing new coagulopathies in the future. The lack of research in this area is also reflected in that the optimal dose or time interval for vitamin K administration is unknown. In a previous study, wherein repeated intravenous doses of 10 mg of vitamin K were given to ICU patients, the largest delta change was seen after two consecutive doses, whereas a third dose was less effective [23]. A 10 mg dose/week has been recommended for critical care patients with a bleeding risk by Hunt in a previous review, and the author reflects over the lack of research of vitamin K supplementation in intensive care [24]. However, for patients who need to go back on treatment with vitamin K antagonists such as warfarin, repeated doses of vitamin K should not be given as it could delay the anticoagulative effect for several days [7].

The clinical significance of a spontaneously increased PT-INR in a non-bleeding critical care patient is unclear [25]. Further, many patients with a spontaneously increased PT-INR receive prophylactic treatment with plasma before an invasive procedure, but with low evidence [5, 6]. Even though PT-INR can be normalized, the haemostatic balance measured with thrombin generation and thromboelastography is usually not normalized [26]. Having a PT-INR in the range < 1.8 does not generally put the patient at risk for clinically significant bleeding events, as this level corresponds to coagulation factor levels > 30% [27]. However, before epidural or spinal anaesthesia, a PT-INR < 1.5 is usually aimed at [28], and in patients with risk for haemorrhagic progression of cerebral contusions, a prolonged PT-INR is one of several risk factors for progression [29]. Recent Nordic guidelines on severe traumatic brain trauma recommends a PT-INR below 1.3 [30]. Trauma-associated coagulopathy has an increased mortality at PT-INR > 1.5, but the relevance of PT-INR 1.2–1.5 is unclear [31]. Intravenous vitamin K correction of prolonged PT-INR in trauma patients without anticoagulation has not been addressed so far in research publications. Based on the results in the present study, the administration of vitamin K might be considered as an alternative to plasma to correct prolonged PT-INR before non-urgent surgery and invasive procedures.

There are general limitations associated with a registry-based design, such as the lack of randomization, which increases the risk of bias and might result in groups that are not comparable. Although we used propensity score matching to address this and reduce inter-group variability, the presence of any undetected variable affecting outcome cannot be ruled out. In addition, there were also study-specific limitations. For instance, exclusion criteria that are related to transfusions were only applicable to the time interval between pre- and post-treatment samplings, and they did not consider whether the patients were given plasma, platelets or an erythrocyte concentrate in close proximity to the pre-treatment PT-INR. Although 24 h are considered enough to reach significant increase in vitamin K-dependent coagulation factors [8], it is unclear whether the timing interval used in the study is optimal as the vitamin K-dependent coagulation factors have different rates of synthesis.

## Conclusion

In non-bleeding patients admitted to the ICU with a PT-INR in the range 1.3–1.9, the administration of intravenous vitamin K resulted in a slightly larger decrease in PT-INR compared to controls. Future studies should focus on identifying which patient populations may benefit most from vitamin K administration as well as whether vitamin K could be a better alternative than plasma or prothrombin complex concentrate to improve PT-INR before non-emergent invasive procedures.

## Data Availability

All original data will be available upon reasonable request.

## Abbreviations

APTT: Activated partial thromboplastin time
CRP: C-reactive protein
EMR: Expected mortality rate
ICU: Intensive care unit
IQR: Interquartile range
PCC: Prothrombin complex concentrate
PIVKA-II: Proteins induced by vitamin K absence for factor II
PT: Prothrombin time
PT-INR: Prothrombin time, international normalized ratio
SAPS 3: Simplified acute physiology score
